# Assessment of community led total sanitation and hygiene approach on improvement of latrine utilization in Laelay Maichew District, North Ethiopia. A comparative cross-sectional study

**DOI:** 10.1371/journal.pone.0203458

**Published:** 2018-09-07

**Authors:** Brhane Gebremariam, Gebremedhin Hagos, Mebrahtu Abay

**Affiliations:** 1 Departement of Public Health, College of Health Sciences, Aksum University, Aksum, Ethiopia; 2 Departement of Biomedical Science, College of Health Sciences, Aksum University, Aksum, Ethiopia; University Lyon 1 Faculty of Dental Medicine, FRANCE

## Abstract

**Background:**

Lack of latrines remain a widespread health and environmental hazard in many developing countries. Low latrine utilization mostly affects the poor, rural and marginalized communities as the majority of those who do not use improved latrines live in rural areas where 90% of all open defecation takes place. The counterpart to this problem, Community-Led Total Sanitation, and hygiene (CLTSH) is an approach that involves facilitating a process to inspire and empower communities to stop open defecation and to build and use latrines in a participatory manner.

**Objective:**

This study was aimed at assessing the Community Led Total Sanitation and Hygiene approach on improvement of latrine utilization in Laelay Maichew District of Central Zone, Northern Ethiopia.

**Methods:**

A comparative cross-sectional study was conducted in Laelay Maichew District of Central Zone, Northern Ethiopia from November 2016 to January 2017. The study subjects were randomly selected 388 households from CLTSH implemented kebeles and 388 households from CLTSH non-implemented kebeles. Systematic random sampling technique was used to select households among proportionally allocated sample frame of households. Then, Interview of household heads using semi-structured questionnaire was conducted to collect data. Finally, data were entered and analyzed using SPSS version 20.0. Bivariate Logistic regressions model was used to identify candidates of multiple logistic regressions. Those P-values < 0.25 were considered as candidates to multiple logistic regressions to determine independent factors of latrine utilization. Variables with Odds Ratio at 95% CI and P-value < 0.05 was considered statistically significant. The study obtained approval from Aksum University Institutional Review Board before its commencement.

**Result:**

This study indicated that the level of latrine utilization and latrine availability in CLTSH implemented kebeles were greater than that of CLTSH Non-implemented kebeles. The finding of this study revealed that the rate of latrine utilization in the rural community of Laelay-matches district was about 47.4%, 95% CI (42.9%-51.8%). The majority (71.1%) of household in CLTSH implemented kebeles and (93.5%) of households in CLTSH non-implemented kebeles did not have hand washing facility near the latrine. Households which had no fresh excreta in around latrine were significantly 11.5 times higher than **[AOR: 11.5, 95% CI (0.18, 50.2)]** utilizing their latrine in CLTSH implemented kebeles.

**Conclusion:**

The study showed that the level of latrine utilization in CLTSH implemented and that of CLTSH non-implemented kebeles was low. Therefore, concerted efforts should be made by local and national governmental and non-governmental organization to should be used to promote behavioral change in the communities to implement community-led total sanitation and hygiene for improving latrine utilization.

## Introduction

### Background

Poor sanitation remains a major threat to development, impacting countries’ progress in health, education, gender equity, and social and economic development worldwide. Globally, 2.5 billion people do not use improved sanitation; 1.2 billion, practice open defecation and 83 percent of whom live in 13 countries in which Ethiopia is one of them accounting for 52 million people. People in rural areas, children, Women, adolescent girls, children, and infants suffer most from inadequate hygiene and sanitation facilities[1, 2]. Moreover, having better water and sanitation is essential to breaking the cycle of poverty since it improves people’s health, strength to work and ability go to school using different approaches [2].

Each year, 200 million tons of human waste goes uncollected and untreated around the world and an estimated 1.5 million death of children under the age of five,5 billion productive days lost, 443 million school days lost are attributed to diarrheal disease globally [3].

Considering the devastating consequences of poor sanitation, in recent years sanitation programs including Community-Led Total Sanitation and Hygiene (CLTSH) have evolved dramatically most of them in which focused on engaging communities, creating demand for sanitation, and supporting the development of sustainable systems and appropriate technologies in which all of which are rooted in catalyzing community behavior and social change [4].

CLTSH is based on the principle of triggering collective behavior change with basic principles of no toilet subsidy and no financial reward when the community reaches 100% Open Defecation Free (ODF). In this approach, communities are simply facilitated to take collective action to adopt safe and hygienic sanitation behavior and ensure that all households have access to safe sanitation facilities [5]. In the process, the community is sensitized to the consequences of poor sanitary practices, commits itself to find own solutions, and finally is liberated from open defecation. This helps to increase a receptive environment for the adoption of improved practices in personal hygiene, safe handling of food and water as well as safe confinement and disposal of excreta and waste[4, 6, 7].

Assuming this recently implementation of CLTSH, Ethiopia’s expansion on CLTS to include hygiene component, is whereby basic hygiene behaviors, including hand-washing with soap (or ash) and water at critical times, and safe water handling and treatment at the household level, are also addressed along with the drive to achieve ‘open defecation free’ status in all villages of the country. it has been started since 2006with a vision of 100% improved household and institutional hygiene and sanitation and expected to facilitate in termination of open defecation through consistent latrine utilization in the country as 60% of overall diseases in Ethiopia which is related to poor sanitation and lack of hygiene [8].

Community-led total sanitation and hygiene approach implementation were started in different parts of Ethiopia; nevertheless, the assessment of CLTSH approach on the utilization of latrine was not assessed, particularly in the study area. Therefore, the objective of this study is to assess the Community-led Total Sanitation and hygiene approach on the improvement of latrine utilization in the rural community of Laelay Maichew district, Central zone, Tigray, Northern Ethiopia.

## Methods and materials

### Study design and period

In this study community based comparative cross-sectional study design was employed. The study was conducted in Laelay Maichew District from November 2016 to January 2017 in Central Zone Tigray, Ethiopia.

### Source and study population

All households in the kebeles of Laelay Maichew District were the source population. Randomly selected kebeles in the district and sampled households was the study population. Sample size determination and sampling techniques.

The sample size was calculated assuming two population proportion formula using rate of latrine utilization 50% for the two study sets, 95% Confidence level, 5% margin of error and 10% of non-response rate. The study subjects were selected using multistage sampling procedure, where the kebeles first divided into community-led total sanitation and hygiene implemented and community-led total sanitation and hygiene non-implemented, then three kebeles were selected from each total kebelles by lottery method. Then, to draw a sampling frame the total number of households in the kebelles were obtained from the respected Health Extension workers of each kebele. The sample size was allotted to each selected kebele by probability proportional to size sampling method. Using systematic random sampling every 9^th^ household)in the selected kebelles were included in the study. The final sample size included in the study was 776 (388 from CLTSH implemented and 388 from CLTSH non-implemented kebeles).

### Data collection method and instrument

Data was collected using a structured questionnaire prepared by reviewing previously done studies and other materials related to the topic. Data were collected through interview followed by latrine observation after the interview (**S1 Questionnaire**).

### Data quality control

To maintain the quality of data, training of data collectors, pre-testing of questionnaire and translation to local language were made. Regular and continues to follow up was made by the principal investigator to monitor the quality of the data collection process and every filled questionnaire was checked on a daily basis and feedbacks were given to data collectors.

### Variables

#### Dependent variable

- Latrine utilization

#### Independent variable

- Socio-demographic & economic variables: Age, religion, marital status, educational level, house hold income, residential setting.
- Behavioral factor variables: frequency of latrine use, Latrine use by ≥ five years old, Observable feces in the compound and latrine, Disposal means of feces of children.
- Environmental factor variables: Latrine availability, Place of defecation, years of latrines construction, Presence of Squat hole cover, maintenance of superstructure, the location of hand washing facilities near the latrine, a distance of latrine from the house and dwelling and Frequency of visit by local leaders.

### Data processing and analysis

The collected data were coded, checked for errors, cleaned and corrected for errors and entered by using SPSS version 20.0 and analyzed. Binary logistic regression was done to determine whether the independent variables can predict the outcome variable. The result of the odds ratio was used for interpretation of the strength of prediction of the independent variables to the outcome variable. The finding from all analysis was summarized and presented in graphs, tables, and other summary measures. For all statistical significance tests, a cut-off value of CI 95% and p<0.05 was used.

### Ethical consideration

Ethical clearance was obtained from the ethical review Board of Aksum University College of Health Sciences. Letter of Permission was obtained from the District health office as well as from each kebele administration. During data collection, verbal and written consent was also obtained from study participants after the purpose of the study was explained.

### Operational definitions

Community-Led Total Sanitation and Hygiene (CLTSH): Emphasizes changing sanitation and hygiene behavior of communities towards open defecation free environment, hand washing practice and keeping drinking water safe.

Latrine Utilization: latrine was considered to be utilized, when every member of the community uses latrine.

## Results

### Sociodemographic characteristics of respondents

Overall, a total of 776 study respondents participated in the study with a response rate of 100%. The mean ± standard error (S.E) age of the respondents was 41.92 ± 5.45 years with an average household family size of 5.51 (**Table 1**).

**Table 1:** Socio-demographic characteristics of respondents conditions of the study households of the study site, Laelay Maichew District, September 2017.

Variable		Status of CLTSH Implementation	
		CLTSH Implemented No (%)	CLTSH Non-implemented No (%)
Sex	Male	249(64.2)	259(66.8)
	Female	139(35.8)	129(33.2)
Age	18–25	6(1.5)	3(0.8)
	25–40	172(44.3)	188(48.5)
	>40	210(54.1)	197(50.8)
		
Marital status	Married	349(89.9)	384(99)
	Single	13(3.4)	3(0.8)
	Widowed	11(2.8)	0(0)
	Divorced	15(3.9)	1(0.3)
Educational status	Illiterate	187(48.2)	154(39.7)
	Grade 1-4^th^	185(47.7)	216(55.7)
	Grade 5-12^th^	13(3.4)	18(4.6)
	Diploma and above	3(0.8)	0
Monthly Income	< 350	106(27.3)	120(30.9)
	350–550	118(30.4)	148(38.1)
	551–750	69(17.8)	42(10.8)
	>750	95(24.5)	78(20.1)
Household head responsibility	Farmer	361(93)	382 (98.5)
	Daily laborer	12(3.1)	2(0.5)
	Merchant	15(3.9)	4(1)
Presence of school children	No	67(17.3)	108(28.1)
	yes	320(82.7)	276(71.9)
Family size	1–3	44(11.3)	10(2.6)
	4–6	246(63.4)	250(64.4)
	>7	98(25.3)	128(33)

### Environmental characteristics

Two hundred sixty-four (68%) households in CLTSH implemented kebeles and two hundred thirty (59.3%) households in CLTSH non-implemented kebeles had latrine facility. Sixty-eight (55.3%) & seventy-seven (48.7%) of households in CLTSH implemented and non-implemented kebeles which have no latrine, practices open defecation respectively. One hundred eighty-five (70.1%) of households in CLTSH implemented kebeles and one hundred sixty-four (71.3%) of households in CLTSH non-implemented kebeles had been constructing their latrine before two years respectively. One hundred twenty-nine (49%) of households in CLTSH implemented and one hundred twenty-nine (50%) in CLTSH non-implemented kebeles need maintenance and repair respectively. One hundred sixty-two (61.4%) of latrine in CLTSH approach implemented kebeles and two hundred thirty-one (57%) of latrine in CLTSH approach non-implemented kebeles were do not have cover for latrine squat hole.

About one hundred eighty-eight (71.2%) of latrines in CLTSH implemented kebeles and one hundred ninety-five (84.8%) of latrines in CLTSH non-implemented kebeles were six to ten meter far away from dwelling room. Two hundred five (53%) of households in CLTSH implemented kebeles and two hundred ten (54.3%) households in CLTSH non-implemented kebeles took less than thirty minutes to the nearest health institution.

Furthermore, two hundred forty-nine (64.2%) of households in CLTSH implemented kebelles and two hundred twenty-one (57.9%) households in CLTSH non-implemented kebelles had near to medium distance to their respected kebelles. Two hundred fifty-three (95.8%) of households in CLTSH implemented kebelles and two hundred fifteen (93.5%) of households in CLTSH non-implemented kebelles have no any hand washing facility near their latrine respectively (**Table 2**).

**Table 2:** Environmental conditions of households of the study site, Laelay Maichew District, September 2017.

Variables		Status of CLTSH Implementation	
		Implemented Kebelles No (%)	Non-implemented Kebelles No (%)
Latrine availability (n = 776)	No	124 (32)	158(40.7)
	Yes	264(68)	230(59.3)
Place of defecation	Open field	69(55.6)	77(48.7)
	Other	55(44.4)	81(51.3)
Year of construction	< 2 years	79(29.9)	66(28.7)
	> 2 years	185(70.1)	164(71.3)
Latrine need repair and maintenance	Yes	129(49)	129(50)
	No	134(51)	101(49)
Presence of squat hole cover	No	162(61.4)	131(57)
	Yes	102(38.6)	99(43)
Presence of hand washing facility near latrine	Present	11(4.2)	15(6.5)
	Absent	253(95.8)	215(93.5)
Distance from the nearest health institution	< 30 min	206(53.1)	211(54.4)
	>30 min and above	182(46.9)	177(45.6)
Distance of latrine to dwelling room	<6 meter	40(15.2)	18(7.8)
	6–10 meter	188(71.2)	195(84.8)
	>10 meter	36(13.6)	17(7.4)
Distance of home from kebelle	Nearest to medium	249(64.2)	223(57.5)
	Far	139(35.8)	165(42.5)
Frequency of visit by local leaders per week	Once	38(9.8)	83(21.4)
	Twice	148(38.1)	146(37.6)
	Three and above	202(52.1)	159(41.0)

### Behavioral factors

Of the respondents, one hundred forty-five (54.9%) of households in CLTSH implemented kebelles and eighty-nine (38.7%) households in CLTSH non-implemented kebelles utilize their latrine. One hundred eighty (71.1%) of the CLTSH implemented and two hundred one (93.5%) of CLTSH non-implemented kebelles households believe that hand washing facility near latrine is not necessary. One hundred forty-seven (37.9%) in CLTSH implemented kebelles and one hundred seventy-seven (45.6%) of CLTSH non-implemented kebelles households do not wash their hand after using the toilet. One hundred thirty-two (34%) in CLTSH implemented kebeles and one hundred one (26%) of CLTSH non-implemented kebelles households got less than 10L/C/ day average water consumption. Ninety-five (39.4%) in CLTSH implemented kebelles and eighty-one (38.4%) of CLTSH non-implemented kebelles of the households always wash their hands. In one hundred seventy (64.9%) in CLTSH implemented kebelles and one hundred twenty-six (54.3%) of CLTSH non-implemented kebelles households had observed fresh excreta around the latrine **(Table 3).**

**Table 3:** Behavioral conditions of the study households of the study site, Laelay Maichew District, September 2017.

Variables		Status of CLTSH Approach Kebeles		
		Implemented No (%)	Non-implemented No (%)	
Latrine utilization (n = 494)	Yes	145(54.9)	89(38.7)	
	No	119(45.1)	141(61.3)	
Hand washing practice after using the toilet (= 776)	Yes	241(62.1)	211(54.4)	
	No	147(37.9)	177(45.6)	
Number of hand washing (N = 452)	Always	95(39.4)	81(38.4)	
	Sometimes	146(60.6)	130(61.6)	
Water consumption per person per liter (= 776)	≤10 liter	132(34)	101(26)	
	>10 liter	256(66)	287(74)	
Presence of feces around the latrine (n = 494)	Yes	92(35.1)	106(45.7)	
	No	170(64.9)	126(54.3)	
Presence of fresh excreta in the compound (n = 494)	Yes	131(33.8)	207(53.4)	
	No	257(66.2)	181(46.6)	

The above figure indicates, 36.7% of latrines of household in CLTSH implemented kebelles and 48.1% of latrines in the households in CLTSH non-implemented kebeles with the availability of latrine needs super structural maintenance (**S1 Fig**).

The above figure indicated that 71.1% of households in CLTSH implemented kebeles and 93.5% of household heads in CLTSH non-implemented kebelles perceive that constructing hand washing facility near latrine is either not necessary or they use alternative to hand washing facility at home (**S2 Fig**).

### Factors associated with latrine utilization

In the multivariable logistic regression model, variables which were significantly associated in the bivariate analysis were re-evaluated independently controlling for other potential confounders. Distance from health facility, distance of kebelle from house, Distance from dwelling, presence of fresh excreta, frequency of visit by health professionals and family income in the CLTSH implemented kebelles and latrine year of construction and distance of latrine from dwelling in the CLTSH non-implemented kebelles were remained to be independent associated factors of latrine utilization.

Households owned latrines for < 2 years were utilizing 3.48 more likely **[AOR = 3.48, 95% CI: (5.40, 24.52)]** than those of owning latrines for more than two years in CLTSH implemented kebelles. Latrine year of construction was not significantly associated in CLTSH non-implemented kebelles.

Households which had no fresh excreta around their latrine were significantly 11.5 times higher than **[AOR: 11.5, 95% CI (0.18, 50.2)]** utilizing their latrine in CLTSH implemented kebelles. Households which were visited three times a week by health professionals in CLTSH implemented kebelles had 2.48 times more likely **[(AOR = 2.48, 95% CI (1.14, 5.39)]** and 2.56 more likely **[AOR = 2.56, 95% CI (1.55, 4.23)]** to utilize their latrine than those of visited once a week. Furthermore, the odds of utilizing latrine in households with income of above 750 ETB were 2.1 times higher than **[AOR: 2.1, 95% CI (1.20, 3.82)]** those who have below 750 ETB in CLTSH implemented kebelles. The odds of utilizing latrine in households with less than ten meter latrine distance from dwelling were 8.5 times higher than **[AOR: 8.5, 95% CI (1.29,56.5)]** those who have greater than 10 meter distance in CLTSH non-implemented kebelles. But Frequency of visit by Health Professionals, presence of fresh excreta and family income in birr was not significantly associated in CLTSH non- implemented kebelles (**Table 4**).

**Table 4:** Multivariate regression of the relative effect of variables on latrine utilization, Laelay Maichew District, September 2017.

Variables	Category	Status of Community Led Total Sanitation and Hygiene									
		Implemented kebelles					Non-Implemented Kebelles				
		Not-Utilized		Utilized		AOR (95% CI)	Not-Utilized		Utilized		AOR (95% CI)
		№	%	№	%		№	%	№	%	
Year of latrine construction	< 2 year	40	50.6	39	49.4	3.48(1.21,10.1)*					
	>2 year	106	57.3	79	42.7	1					
The distance of latrine from dwelling	<10 meter						3	16.7	15	83.3	8.5(1.29,56.5)*
	6–10 meter						76	39.0	119	61.0	2.1(0.65, 6.81)
	>ten meter						10	58.8	7	41.2	1
Frequency of visit by HP	Once a week	24	63.2	14	36.8	1					
	Twice a week	126	62.4	76	37.6	2.48(1.14, 5.39)*					
	Three times a week	119	81.0	28	19.0	2.56(1.55, 4.23)*					
Presence of fresh excreta around the latrine	No	93	54.7	77	45.3	11.5(5.40,24.52)*					
	Yes	53	53.0	39	47.0	1					
Family income in birr	< 350	59	55.7	47	44.3	1					
	350–550	89	76.1	28	23.9	0.83(0.45,1.53)					
	551–750	52	75.4	17	24.6	0.87(0.43,1.76)					
	> = 750	69	72.6	26	27.4	2.1(1.20,3.82)*					

* = significantly associated at p<0.05

## Discussion

The findings of this study revealed that the rate of latrine utilization in the study area was was 47.4%, 95% CI (42.9% - 51.8%) which was slightly lower than a study conducted in Gulemokada district which was 57.3%[9] and Hulet Ejju Enessie district, East Gojjam Zone, Amhara Region 60.7%[10] and Alaba and Mirab Abaya districts 93%, Ethiopia[11]. The possible reason for the low utilization rate might be due to decentralized managing system and low political priority of hygiene and sanitation in the study area. Latrine utilization rate of this study is also comparable with 2011 baseline survey report on WASH, which was 34%[12] and Hawzien district 37.3% in Tigray region [13]. The disparity might be attributed to the better involvement of Non-Governmental Organizations (NGO’s) and governmental interventions in the study area. In the present study, there is no organized and continuous implementation of Community Led Total Sanitation and Hygiene transformation intervention except the provision of advice and education by health extension workers, local administrators and local NGO’s (Relief of Society of Tigray). The low use of latrines in the study area can be explained that health extension workers promote the construction of latrine rather than utilization and less active in teaching proper latrine utilization.

This study showed that households owned latrines for more than two years were utilizing more likely [(AOR = 8.5 95% CI (1.29, 56.5)] than owning latrines less than two years. This may be attributed to the perception of the community to gain immediate health benefit like cleanliness and reduction of fly breeding. Whereas, it had no effect on households which did not implement CLTSH.

In this study, the only variable significantly associated with latrine utilization of households in CLTSH non-implemented kebeles was a distance of latrine from the dwelling. This may be due to familiarity and awareness of the community in the study area about CLTSH which was incompletely and disorganized given by some local NGO’s like Relief Society of Tigray (REST). This study was in similar with a study done in Bahir Dar Zuria, Ethiopia [14].

The findings of this study also showed that the households lacked latrines, about 55.3% of households in CLTSH implemented and 48.7% of households in CLTSH non-implemented kebeles were practiced open defecation during the survey. This was similar to a study done in Kersa District, Jimma zone, Ethiopia [15].

In this study, almost half of the latrines in both CLTSH implemented (49%) and (50%) in CLTSH non-implemented kebelles needs maintenance and repair. The presence of a handwashing facility near latrine encourages the users to wash their hands after latrine use [15]. However, in multivariate analysis, latrine provided with hand washing facility was not significantly associated in both CLTSH implemented and non-implemented. Ninety-six percent in CLTSH implemented and 93.5% in CLTSH non-implemented kebeles have no hand washing facility near their latrine. In a study done in Gulomekada [9] the presence of school children was associated with latrine utilization, whereas in our study this variable was not significantly associated.

## Conclusions

The study showed that the extent of latrine utilization in CLTSH implemented was greater than that of CLTSH non-implemented kebeles. In this study coverage of hand-washing facility near the latrines in both CLTSH implemented kebeles and CLTSH non-implemented kebeles was very low. The study also indicated that from those households with latrine the habit of hand-washing after defecation in CLTSH implemented and non-implemented kebelles was similar and have no significant association in the bivariate and multivariate analysis in both kebelles. Thus, even though the CLTSH implementation seems almost the same in both the CLTSH implemented and non-implemented kebelles, it can be concluded that it is possible to increase latrine utilization rate through the effective and sustainable implementation of the CLTSH approach. Therefore, it is possible to increase latrine utilization with the sustainable implementation of the community-led total sanitation and hygiene approach.

## Supporting information

S1 Fig Part of latrine which needs maintenance and repair of the study area, Laelay Maichew District, September 2017. (TIF)

S2 Fig Reasons for not constructing Hand washing facility near latrine of the study area, Laelay Maichew District, September 2017. (TIF)

S1 Questionnaire An English and a Tigrigna version questionnaires. (PDF)

S1 Data The minimal anonymized dataset used for analyses. (SAV)

## Abbreviations

CLTSH: Community Led Total Sanitation and Hygiene
FMOH: Federal Ministry of Health
GTP: Growth and Transformation Plan
MDG: Millennium Development Goal
NGO’s: Non-Governmental Organizations
ODF: Open Defecation Free
SNNPR: Southern Nation’s Nationalities and Peoples Region
UNICEF: United Nation’s International Children’s Emergency Fund
