# Supplementary figures and images for: Assessment of community led total sanitation and hygiene approach on improvement of latrine utilization in Laelay Maichew District, North Ethiopia. A comparative cross-sectional study
Source: PLoS One. 2018 Sep 7;13(9):e0203458. doi: 10.1371/journal.pone.0203458 (PMC6128552; doi:10.1371/journal.pone.0203458)

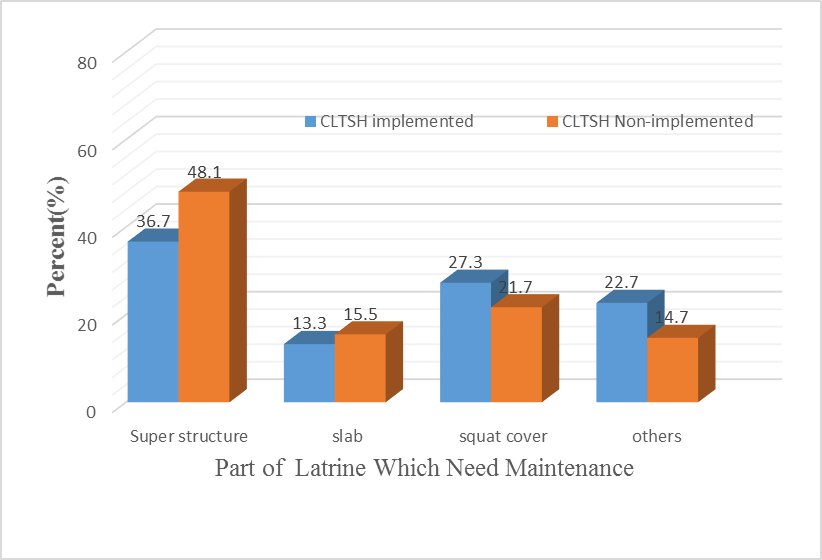

Supplement: S1 Fig — (TIF) [file pone.0203458.s001.tif]

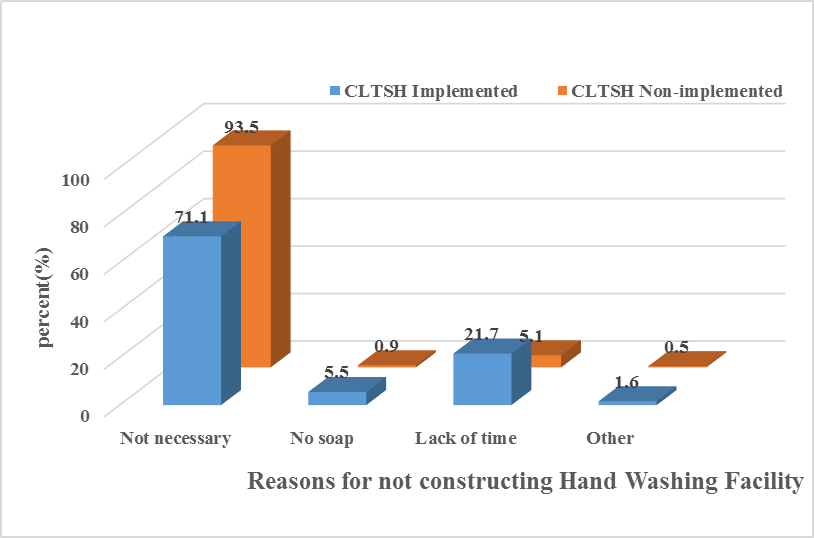

Supplement: S2 Fig — (TIF) [file pone.0203458.s002.tif]
