# Supplementary material for: Assessment of community led total sanitation and hygiene approach on improvement of latrine utilization in Laelay Maichew District, North Ethiopia. A comparative cross-sectional study
Source: PLoS One. 2018 Sep 7;13(9):e0203458. doi: 10.1371/journal.pone.0203458 (PMC6128552; doi:10.1371/journal.pone.0203458)
Supplement: S1 Questionnaire — (PDF) [file pone.0203458.s003.pdf]

CONSENT FORM: **Consent to participate in Research Study**

Title of Study: Implementation of CLTSH on latrine utilization in Laelay Maichew district.

Principal Investigator: Brhane Gebremariam (MSc in Environmental Health), +251912806855

Email: gebremariambrhane@gmail.com

Principal Investigator Department: Public Health

My name is ______________________.you are being asked to take part in a research study. To join the study is voluntarily. You may refuse to join, or you may withdraw your consent to be in the study, for any reason, without penalty. You may not receive any direct benefit from being in the research study. There are no risks being in this research study. It is important that you understand this information so that you can make an informed choice about being in this research study. You should ask the researcher named above any questions you have about this study at any time. The investigator named above is highly interested to assess implementation of Community led total sanitation and hygiene approach on latrine utilization in Laelay Maichew District. The results you give us will help us find out the present conditions in your community. A total of a 776 will be included in the study from Laelay Maichew district. The total length of interview and observation will be around 30 minutes. Your privacy and confidentiality will be stored separately in a secure location, and names were coded to protect privacy. The investigator has the right to stop your participation in the unlikely event that the entire study has been stopped. You will not receive a monetary incentive for your participant in this study.

Participant's Agreement:

I have read the information provided above. I have asked all the questions I have at this time. I voluntarily agree to participate in this research study.

Signature of Person administering consent__________ Interviewer (Name &Number):_____Date___

Date of interview: Day ______ Month ________ Year_______

**Part I. Socio-Part I. Socio-economic characteristics**			
**No.**	**Questions**	**Alternative responses**	**Skip to**
101	Sex of the respondent	1. Male<br>2. Female	

102	Age of the respondent in years?	____	
103	What is your religion	1. Orthodox<br>2. Catholic<br>3. Protestant<br>4. Muslim<br>6.Others (specify)______	
104	What is your current marital status??	1.Single<br>2. Married<br>3. Divorced<br>4. Separated<br>5. Widowed	
105	What is the highest level of school you have completed?	1.Cannot read and write<br>2. Grade_____<br>3. Diploma and above	
106	What is your average monthly income?	_______	
107	Head of household	1. Male<br>2. Female<br>3. Other (specify)	
108	Occupational status of household head	1. Farmer<br>2. Daily Labor<br>3. Merchant<br>4. Others (specify)	
109	Number of household members	_________	
110	Do you have children attending school	1. Yes<br>2. No	
**Part II. Environmental Factors**			
201	Do you have latrine	1. Yes<br>2. No	
202	When was your latrine constructed?	1. <2 year<br>2. ≥2 year	

203	Main source of information for latrine construction	1. Health professionals<br>2. Through sanitation campaign<br>3. Family members<br>4. Mass media<br>5. Neighborhoods<br>6. students	
204	Is Latrine squat covered	1. yes<br>2. No	Observation
205	Does latrine need maintenance currently?	1. Yes<br>2. No	Observation
206	If yes for Q205, Part of latrine which needs maintenance	1.Super structure<br>2.Slab<br>3.Roof<br>4. Mixed<br>5.Other (Specify___)	
207	Did you use your latrine	1. Yes<br>2. No	Observation
207	Walking time from home to health institution?	1. < 30 minutes<br>2. ≥ 30 minutes	
208	Distance of Latrine from home?	1. < 6 meters<br>2. 6-10 meters<br>3. >10 meters	
209	Keble accessibility	1. Near to medium<br>2. Too far	
210	Frequency of supervision by health professionals	1. 1-2 times per months<br>2. times per month<br>3. More than three times per month	
211	Frequency of supervision by local leaders	1. Once a week<br>2. Twice a week	

		3. three times a week	
212	Availability of Hand washing facility	1. Yes<br>2. No	IF yes, skip to number 213
213	Detergent used for Hand washing?	1. Nothing<br>2. Soap<br>3. Ash	Observation
214	Distance from water source in Meter?	____	
**Part III. Behavioral Factors**			
301	Who motivate you to construct latrine?	1. Self -initiation<br>2. Kebele leaders<br>3. Primary health workers<br>4. Others	
302	If no latrine why not	1. Too expensive<br>2. Nearest toilet here<br>3. No land to build one<br>4. There are pests	
303	If no latrine Where do you defecate	1. Open field<br>2. Others (specify)	
304	Hand washing after using toilet	1. Yes<br>2. No	
305	Detergent used for hand washing	1. Only water<br>2. Soap<br>3. Ash<br>4. Others (specify)…	
306	Frequency of hand washing a	1. Always<br>2. At all critical time/some times	
307	Average water consumption in L/per/day	1. <10<br>2. 10– 20	

308	Place of children feces disposal	1. In the open field<br>2. Others	
309	Frequency of latrine use	1. Always<br>2. Sometimes	
310	Observable faeces in the compound	1. Yes<br>2. No	
311	Presence of fresh faeces around the latrine	1. Yes<br>2. No	
312	Extent of latrine utilization	1. Satisfactory<br>2. Unsatisfactory	
313	Starting age of latrine use by family members	1. At 2 years old<br>2. At 3 years old<br>3. At 4 years old	
314	Latrine use by under-five children	1. Yes<br>2. No	
315	Fresh feces around the squat hole	1. Yes<br>2. No	

ጥዕና ሳይንስ ኮሌጅ

ትምህርቲ ክፍሊ ሕብረተሰብ ጥዕና

<u>እንታይነት መረዳእታ ናይ ሓታትን ፍቓድ ስምምዕነት ቅጥዕን ዝሕብር ማሕተት</u>

ሽመይ ___________________ይበሃል:: ኣነ ኣብ ኣክሱም ዩኒቨርስቲ ትምህርቲ ክፍሊ ሕብረተሰብ ጥዕና መምህር እየ::

እትሕተት/ቲ ዘለኻ/ኺ ኣብ ምርምር መፅናዕቲ ንኽትሳተፍ/ፊ እዩ፡፡ ርእሲ እዚ ምርምር "ገምጋም ሕ/ሰብ ዝመርሖ ከባብያውን ዉልቃዉን ፅረት" ዝብል እዩ፡፡ ኣብዚ መፅናዕቲ ንምስታፍ ድሌት መሰረት ዝገበረ እዩ፡፡ ኣብዚ መፅናዕቲ ዘይምስታፍን ብዝኾነ ይኹን ምኽንያት ብዘይ ምንም ቅፅዓት ስምምዕነት ምቁራፅን ትኽእል/ሊ ኢኻ/ኺ፡፡

ምርምር መፅናዕቲታት ሓዱሽ ፍልጠት ንምርካብ ዝተቐረፁ እዮም፡፡ እዚ ሓዱሽ መረዳእታ መፃኢ ንህዘቢ ክሕግዝ ይኽእል እዩ፡፡ ኣብ እዚ መፅናዕቲ ብምስታፍካ/ኪ ቀጥታ ጠቐሚ ዘይክትረክብ/ኽቢ ትኽእል/ሊ ኢኻ/ኺ፡፡ ኣብ እዚ መፅናዕቲ ብምስታፍካ/ኪ ጉድኣት የብሉን፡፡ ብዛዕባ እዚ መፅናዕቲ ኣብ ታሕቲ ብዝርዝር ቀሪቡ እዩ፡፡ እዚ ሓበሬታ ምርዳእኻ/ኺ ጠቓሚ እዩ፡፡ ስለዚ ብዛዕባ ኣብዚ መፅናዕቲ ምስታፍ ክትመርፅ/ፂ ትኽእል/ሊ ኢኻ/ኺ፡፡ እትህብዎ ሓበሬታ ንመንግስትን ዝተፈላለዩ ገበርቲ ሰናይ ኣካላትን ናይ ጥዕና ትልሚ ንምዉፃእ ይጠቅም እዩ፡፡እዚቃለመሕትትእዚነዓኹምኮነንቤተሰብኩምዝኾነይኹንጉደኣትከምዘየምፅእከረጋግፀ ልኩምይፈቱ፡፡ካብዚ ብተወሳኺ ናታትኩም ስምን ኣድራሻን ኣይወስድን::ዝኾነ ይኹን እትህብዎ ሓበሬታ ብዘይካ መፅነዓይ ኣካል ሚስጢሩ ዝተሓለወ እዩ፡፡ኣብዚ መፅናዕቲ ንምስታፍ ብድልየትኩም እዩ፤ኣብሞንጎ ደስ ዘይበለኩም ሕቶ ኣንተሃልዩ ናብ ቀፃሊ ሕቶ ሕለፍ በሉኒ::እቲ ቃለ መሕትት ኣብ ደስ ዝበለኩም ግዜ ምቁራፅ ይከኣል እዩ::

ኣብቲ መፅናዕቲ ንምስታፍ ፍቓደኛታት ዲኹም/ክን ? 1. *እወ*

2. *ኣይኮናን*

ናይቲ ቃለ መጠየቕ ውፅኢት 1. *ሙሉእ* 3. *ተጠያቂ ኣካልኣይተረኽበን*

2. *ተጠያቂ ኣካል ኣይተቀበሎን* 4. *ማሕተቱ ሙሉእ ኣይኮነን*

ስምምዕነት ቅጥዒ

ኣብዚ መፅናዕቲ ንክሳተፍ ፍቀደኛ ምዃነይ እንትገልፅ ናይቲ መፅናዕቲ ዓላማ ኣጠቓቕማ ሽንት ቤትን ንኽይጥቀሙ ምክንያትን ዝኮኑ ነገራትን ንምፅናዕ ዝዓለመ ከም ዝኮነን ዝህቦ መልሲ ንካሊእ ጥቅሚ ከምዘይውዕል ኣብ ዝኮነ ይኩን ቦታ ስመይ ከም ዘይግልፅ ፣ ምስታፈይ ብድሌት ዝተመስረተ ምዃነን ክምልሶ ዘይደሊ ሕቶ ክገድፎ ከምዝክእል ከምኡ ውን ብምስታፈይ ምንም ፀገም ከምዘይበፅሐኒ ተረዲኤ እየ::

ናይ ሓታታይ ስም_________________________________ፊርማ __________ዕለት_________

ናይ ሱፐርቫይዘር ስም _________________________ፊርማ__________ ዕለት______

ክፍሊ ሓደ፦ማሕበራውን ኢኮኖሚያዊን ዝምልከቱ ሕቶታት

ተ/ቁ	ሕቶታት	ናይ መልሲ መማረፂታት	ዝለል
101	ፆታ	ሀ, ተባዕታይ	
102	ዕድመ ብዓመት	----------------	
	ሃይማኖት	ሀ. ኦርቶዶክስ<br>ለ. ካቶሊክ<br>ሐ. ፕሮቴስታንት	
103	ናይ ሓዳር ኩነታት	ሀ. ዘይተመርዓወ/ት/መዕበይ/ት<br>ለ. በዓልቲ ሓዳር/በዓል	
104	ናይ ትምህርቲ ደረጃ	ሀ, ዘይተምሃረ/ት<br>ለ ዝተምሃረ/ት -----------	
105	ወርሓዊ አታዊ መጠን ገንዘብ	---------------------------	
106	በዝሒ መራሕቲ ስድራ	-----------------	
107	መራሒ ስድራ	ሀ. አቦ ለ.	
108	ናይ ስራሕ ኩነታት	ሀ. ገባር<br>ለ. መዓልታዊ ስራሕተኛ<br>ሐ. ነጋዴ	
109	በዝሒ መራሕቲ ስድራ	-------------------	
110	ት/ቲ ዝከታተሉ ህናት ኣለዉኹም ዶ?	ሀ. እወ<br>ለ. ኣይፋል	
**ክፍሊ ክልተ:- ከባብያዊ ጥዕናን ተግባራትን ዝምልከቱ ሕቶታት**			
201	ሽንቲ ቤት ኣለኩም ዶ	ሀ. እወ<br>ለ. ኣይፋል	
202	እቲ ሽንቲ ቤት ካብ ዝስራሕ ክንደይ ዓመት ጌሩ	ሀ. < 2 ዓመት<br>ለ. >=ዓመት	
203	ሽንት ቤት ንክትሰርሑ ካብ ዘለዓዕለኩም እንታይ እዩ	ሀ. ጥዕና በዓል ሞያ<br>ለ. ኣብ እዋን ከባብያዊ ፅሬት<br>ሐ. ቤተሰብ	

204	እቲ ኣፍ ሽንት ቤት ሽፋን አለዎ ዶ? /ትዕዝብቲ/	ሀ. እወ<br>ለ. ኣይፈል	
205	እቲ ሽንት ቤት ፅገና የድልያ ዶ?	ሀ. እወ<br>ለ. ኣይፋሉን	
206	ንቁፅሪ 204 መልስኩም እወ እንድሕር ኮይኑ ኣብ ምንታዩ	ሀ. ሱፖር ስትራክቸሩ<br>ለ. ስላብ<br>ሐ. ክዳኑ	
207	እቲ ሽንቲ ቤት ትጥቀምሉ ዶ ?	ሀ. እወ<br>ለ. ኣይንጥቀምን	
208	ርሕቐት ጥዕና ትካል ካብ ገዛ ክንደይ ይኸውን?/ ብሜትር/	ሀ. < 30 ደቒቃ<br>ለ. ≥ 30 ደቒቃ	
209	ርሕቐት ሽንቲ ቤት ካብ መንበሪ ገዛ ክንደይ ይኸውን?/ ብሜትር/	ሀ. < 6 ሜ<br>ለ. 6-10 ሜ<br>ሐ. >10 ሜ	
210	ገዛኩም ካብ ቀበሌኩም ክንደይ የርሕቅ	ሀ. ካብ ቀረባ ናብ ማእከላይ	
211	ቀበሌ ኣመሓደርቲ ኣብ ሰሙን ክንደይ ግዜ ዑደት አካይዶም ዶ ነይሮም	ሀ. ኣብ ሰሙን 1 ግዜ<br>ለ. ኣብ ሰሙን 2 ግዜ	
212	ናይ ጥዕና ሞያተኛታት ዑደት አካይዶም ዶ ነይሮም?	ሀ. እወ<br>ለ. ዑደት አየካየዱን	
213	ንሕቶ 212 መልስኩም እወ እንተኮይኑ ክንደይ ግዜ?	ሀ. 1 - 2 ግዜ ብወርሒ<br>ለ. 2 ግዜ ብወርሒ<br>ሐ. 3ን ልዕሊኡን ግዜ	
214	ሽንት ቤት ምጥቃም ዘይጀመሩ ህፃናት ፍታኖም አበይ ኢኹም ትጉሕፍዎ?	ሀ, አብ ከባቢ እቲ ገዛ<br>ለ, ናብ ሽንት ቤት	
215	አብ ከባቢ እቲ ገዛ ፍታን አሎ ዶ?/ትዕዝብቲ/	ሀ, እወ ለ, የለን	
216	እቲ ሽንት ቤት ክንደይ ዘአክል ኢኹም ትጥቀምሉ?	ሀ, ኩሉ ግዜ ለ, ሓለሓሊፉ	
217	እቲ ሽንት ቤት ፅገና የድልያ ዶ	ሀ. እወ ለ. ኣይፋሉን	

218	መሕፀቢ ኢድ ኣብቲ ሽንቲ ቤት ኣሎ ዶ	ሀ. እወ<br>ለ. ዑደት አየካየዱን	
219	አወ እንተኮይኑ ን216 ኢድ መሕፀቢ እንታይ ትጥቀሙ	ሀ. ምንም ኣይንጥቀምን<br>ለ. ሳሙና<br>ሐ. ሓሙክሽቲ	
**ክፍሊ ሰለስተ፥ ናይ ስነ-ባህሪ ዝምልከት**			
301	ካበይ ኢኹም እቲ ሓበሬታ እትረክብዎ?	ሀ. ካብ ጥሙ ር ጥዕና ቤተ ሰብ ሐ. ሬድዮ<br>ለ. ካብ ተሌቪዝን<br>[illegible]	
302	እቲ ሽንቲ ቤትኩም ክትሰርሑ መን ኣናማኪርኩም	ሀ. በዓልና<br>ለ. ናይ ቀበሌ ኣመሓደርቲ<br>ሐ. ጥዕና በዓል ሞያታት	
303	ሽንት ቤት እንተዘይሃልዩ ንምንታይ	ሀ. ንምስርሑ ክባር እዩ<br>ለ. ኣብ ጎረቤት ስለዘለና<br>ሐ. ቦታ የብልናን	
304	ሽንት ቤት እንተዘይሃልዩ ኣበይ ትጥቀሙ	ሀ. ኣብ ደገ<br>ለ. ካሊእ	
305	ሽንት ቤት ምስተጠቀምኩም ኢድኩም ትሕፀቡ ዶ	ሀ.እወ ለ, የለን	
306	አወ እንተኮይኑ ን---- ኢድ መሕፀቢ እንታይ ትጥቀሙ	ሀ. ምንም ኣይንጥቀምን<br>ለ. ሳሙና<br>ሐ. ሓሙክሽቲ	
307	ክንደይ ጊዘ ትሕፀቡ	ሀ. ኩሉ ጊዜ<br>ለ. ሓደሓደ ጊዜ	
308	ብማእከላይ ኣብ መዓልቲ ክንደይ ሊትሮ ትሰትዩ	ሀ. ‹10ሊትር<br>ለ. 10-20ሊትር	
309	ኣብቲ ከባቢ ርሑስ ፍታን ኣሎ ዶ	ሀ. እወ<br>ለ. ኣይፋል	

310	ኣብቲ ሽንት ቤት ርሑስ ፍታን ኣሎ ዶ	ሀ. እወ<br>ለ. ኣይፋል	
311	ደረጃ ኣጠቃቅማ እቲ ሽንት ቤት እንታይ ይመስል	ሀ. ኣዕጋቢ<br>ለ. ኣዕጋቢ ኣይኮነን	
312	ትሕቲ 5 ዓመት ቆልዑ ሽንት ቤት ይጥቀሙ ዶ	ሀ. እወ<br>ለ. ኣይፋል	
313	እቶም ቤተሰብ ኣብ ከንደይ ዕድመኦም ሽንት ቤት ምጥቃም ጀሚሮም	ሀ. ኣብ 2 ዓመቶም<br>ለ. ኣብ 3 ዓመቶም	
